# Extract from* Periostracum cicadae* Inhibits Oxidative Stress and Inflammation Induced by Ultraviolet B Irradiation on HaCaT Keratinocytes

**DOI:** 10.1155/2017/8325049

**Published:** 2017-03-29

**Authors:** Tsong-Min Chang, Jen-Horng Tsen, Hsuan Yen, Ting-Ya Yang, Huey-Chun Huang

**Affiliations:** ^1^Department of Applied Cosmetology and Master Program of Cosmetic Sciences, Hungkuang University, Taichung, Taiwan; ^2^Department of Nutrition, China Medical University, Taichung, Taiwan; ^3^Department of Medical Laboratory Science and Biotechnology, College of Health Care, China Medical University, Taichung, Taiwan

## Abstract

*Periostracum cicadae* is widely used for the treatment of skin diseases such as eczema, pruritus, and itching. The current study sought to evaluate the effect of* P. cicadae* extract on ultraviolet B (UVB) irradiation and identify the mechanisms involved. Photodamage-protective activity of* P. cicadae* extracts against oxidative challenge was screened using HaCaT keratinocytes.* P. cicadae* extracts did not affect cell viability but decreased reactive oxygen species (ROS) production. The extract attenuates the expression of interleukin-6 (IL-6), matrix metalloproteinase-2 (MMP-2), and MMP-9 in UVB-treated HaCaT cells. Also,* P. cicadae* abrogated UVB-induced activation of NF-*κ*B, p53, and activator protein-1 (AP-1). The downmodulation of IL-6 by* P. cicadae* was inhibited by the p38 inhibitor (SB203580) or JNK inhibitor (SP600125). Moreover, the extract attenuated the expression of NF-*κ*B and induced thrombomodulin in keratinocytes and thereby effectively downregulated inflammatory responses in the skin. The nuclear accumulation and expression of NF-E2-related factor (Nrf2) were increased by* P. cicadae* treatment. Furthermore, treatment with* P. cicadae* remarkably ameliorated the skin's structural damage induced by irradiation. This study demonstrates that* P. cicadae* may protect skin cells against oxidative insult by modulating ROS concentration, IL-6, MMPs generation, antioxidant enzymes activity, and cell signaling pathways.

## 1. Introduction

The traditional Chinese medicine (TCM) therapy in Taiwan has been enrolled in the National Health Insurance Research Database since 2002. The frequently used TCM herbal formulae and single herbs for treating atopic dermatitis (AD) were identified from the electronic medical records [1]. Fang-Feng, Bai-Zhu, and Chan-Tui belonged to the frequent principle of single herbs for atopic dermatitis (AD) patients. Chan-Tui, Latin pharmaceutical,* P. cicadae,* is the most commonly used single herb that has been found to possess many interesting pharmacological and physiological activities, such as antiproliferation [2] and anticonvulsive, sedative, hypothermic, and antioxidant activities [3–5]. In this study, we studied the protective effects of these herbs against UVB and explored the mechanisms of how* P. cicadae* confers protection against UVB in skin. Specifically, the photoprotection effects of* P. cicadae* slough shed on inflammatory tissues were assessed using a mouse model of UVB radiation.

Previous reports have demonstrated that exposure of skin to UVB causes several detrimental skin effects including inflammation [6, 7], immunosuppression [8], premature skin aging, and skin cancer development [9, 10]. At the molecular level, UVB can induce intracellular reactive oxygen species (ROS) generation [11–13], which activates cell signaling and stimulates transcription factor expression including nuclear factor kappa B (NF-*κ*B), activator protein-1 (AP-1), and p53 [14–16]. These transcription factors play important roles in photocarcinogenesis and photoaging. Our previous study demonstrates that UVB irradiation results in ROS accumulation and extracellular signal-regulated kinases (ERKs) activation, which causes the nuclear p53 accumulation and thrombomodulin (TM) promoter binding to inhibit TM expression [17]. To prevent oxidative deterioration, antioxidant extracted from natural herbal sources is widely reported to be used to protect the skin from damage by limiting the production of free radicals, calming inflammation [18], and stimulating blood flow in the skin to encourage the growth of new cells [19, 20]. Nrf2 also plays an important role in protecting the skin against UV irradiation [21]. In unstimulated conditions, Nrf2 is negatively regulated by Kelch-like ECH-associated protein 1 (Keap1), which facilitates the degradation of Nrf2 through ubiquitinated proteasomal degradation [22]. Nrf2 translocates into the nucleus and binds to the promoter region of many genes encoding phase II detoxification enzymes and antioxidants, such as heme oxygenase 1 (HO-1), and NAD(P)H quinone oxidoreductase 1 (NQO1) [23, 24]. The redox-sensitive transcription factor Nrf2 orchestrates major cellular defense mechanisms including phase II detoxification, inflammatory signaling, DNA repair, and antioxidant response, and Nrf2 has therefore emerged as a promising molecular target for the pharmacological prevention of human pathologies resulting from exposure to environmental toxicants including solar UVB. Recent studies strongly suggest a protective role of Nrf2-mediated gene expression in the suppression of cutaneous photodamage induced by solar UV radiation [25–27]. Therefore, pharmacological modulation of Nrf2 has now attracted considerable attention as a novel approach to skin photoprotection [28, 29]. Topical application of Nrf2 inducers could have pronounced photoprotective and photochemopreventive activity against UV-induced human skin erythema. Furthermore, these clinically used herbs might have the potential for application in topical skin care products, which could effectively provide protection against environmental solar damage for AD patients.

The goal of this study was to investigate the effect of* P. cicadae* extract on inflammatory mediators and cellular signaling pathways in UVB-exposed HaCaT cells. The topical application of* P. cicadae* inhibited UVB-induced expression of inflammatory mediators; activation of NF-*κ*B signaling pathways will also be investigated. Furthermore, we also elucidate the effect of* P. cicadae* extract on UVB-mediated protein expression of Nrf-2.

## 2. Materials and Methods

### 2.1. Materials

The herbal formula and single herbs were purchased from Sun Ten Pharmaceutical Co., Ltd. (New Taipei City, Taiwan). The powdered materials (10 g) were then extracted with 50% v/v ethanol (100 mL), with continuous shaking at room temperature for 4 hours with 1 hour at 100°C. The supernatants were centrifuged, filtered, and vacuumed dry and then kept at −20°C. All dried extract was weighed and reconstituted in 0.1% DMSO containing culture medium to 1 mg/mL. Extraction efficiency of each sample was determined by three times extractions. All other chemicals and solvents were obtained from Sigma-Aldrich Inc. (St. Louis, MO).

### 2.2. Cell Culture and UVB Irradiation

HaCaT cells [30] were maintained in DMEM (Hyclone; GE Healthcare Life Sciences, Marlborough, MA) supplemented with 10% fetal bovine serum and 1% antibiotics at standard cell culture conditions (37°C, 5% CO_2_ in a humidified incubator). UVB was generated from a 15 W UVB lamp equipped with an electronic controller at a distance of 30 cm. The UVB dose was calculated accurately with a UVB meter (UVP, Upland, CA). Irradiation doses were calculated using the formula: dose (mJ/cm^2^) = exposure time (s) × intensity (mW/cm^2^). For UVB irradiation, HaCaT cells pretreated with herb extracts for 4 hours were replaced with phosphate-buffered saline (PBS) and irradiated with 12 mJ/cm^2^ UVB (wavelength 290–320 nm). After UV irradiation, fresh media were added to each plate, and cells were maintained in regular culture conditions for 24 hours until analysis.

### 2.3. MTS Assay

The MTS assay (Promega, Madison, WI) was used to assess the effect of UVB on HaCaT cell growth and viability. The number of viable cells was calculated using the MTS colorimetric assay. The medium containing the MTS reagents at a 1 : 10 dilution was added. The cells were incubated for 60 minutes at 37°C and the optical density was measured at 490 nm using a plate reader. Each experiment was repeated at least three times.

### 2.4. ROS Measurement

HaCaT cells were seeded in 96-well plates at a concentration of 1 × 10^5^ cells/mL and were preincubated with herb extracts for 4 hours before UVB (12 mJ/cm^2^) exposure. The UV-irradiated cell cultures were treated with 10 *μ*M 2,7-dichlorofluorescein diacetate (DCFH-DA; Sigma-Aldrich) in PBS for 30 minutes. After incubation, the media were discarded, and the cells were washed with PBS. The cells were monitored by fluorescence microscopy (Olympus IX71) and the fluorescence intensity was determined using a Spectramax M2e fluorescence plate reader (Molecular Devices, Sunnyvale, CA) at 485 nm for excitation and 530 nm for emission. Relative fluorescence intensity was calculated using unexposed control cells as a standard.

### 2.5. Western Blot Analysis

Cells were lysed in PBS containing 1% nonidet P-40, 0.5% sodium deoxycholate, 0.1% sodium dodecyl sulfate (SDS), 5 *μ*g/mL aprotinin, 100 *μ*g/mL phenylmethylsulfonyl fluoride, 1 *μ*g/mL pepstatin A, and 1 mM ethylenediaminetetraacetic acid (EDTA) at 4°C for 20 minutes. Total lysates were quantified using a microBCA kit (Thermo Fisher Scientific, Waltham, MA). Proteins (10 *μ*g) were resolved by SDS-polyacrylamide gel electrophoresis and electrophoretically transferred to a PVDF membrane. The membrane was blocked in 5% fat-free milk in PBST buffer (PBS with 0.05% Tween-20) followed by incubation overnight with the following primary antibodies diluted in PBST buffer: TM antibody (Ab), MMP-2 Ab, JNK Ab, pJNK Ab, p38 Ab, pP38 Ab, c-jun Ab, c-fos Ab, lamin B1 Ab (diluted used in 1 : 1,000, all from Santa Cruz Biotech [Dallas, TX]), MMP-9 Ab (Abcam, Cambridge, UK), p65 Ab, and p53 Ab (Genetex, Irvine, CA). The primary antibodies were removed, and the membrane was washed extensively in PBST buffer. Subsequent incubation with horseradish peroxidase-conjugated goat anti-rabbit antibodies (1 : 20,000, Santa Cruz Biotech) was performed at room temperature for 2 hours. The membrane was washed extensively in PBST buffer to remove any excess secondary antibodies, and the blot was visualized with enhanced chemiluminescence reagent (GE Healthcare).

### 2.6. Q-PCR

RNA samples were reverse-transcribed for 120 minutes at 37°C with high capacity cDNA reverse transcription kit according to the standard protocol detailed by the supplier (Thermo Fisher Scientific). Quantitative PCR was performed as follows: 5°C, 2 minutes; 95°C, 10 minutes; 95°C, 15 seconds; 55°C, 1 minute; 95°C, 15 seconds; 55°C, 1 minute; 95°C, 15 seconds; 60°C, 10 seconds using 2x Power SYBR Green PCR Master Mix (Applied Biosystems, Foster City, CA) and 200 nM of forward and reverse primers. Each assay was run on an Applied Biosystems' 7300 Real-Time PCR system in triplicate. The data from Q-PCR were analyzed with delta-delta threshold cycle (ΔΔCt) method using 18S rRNA as the internal control gene. Each ΔCt value was determined by subtracting 18S Ct value from the target gene Ct value. The ΔΔCt was calculated by subtracting the ΔCt value of the 37°C control from the ΔCt value of the sample. 2^−ΔΔCt^ represented the average relative amount of Nrf-2 or IL-6 for each target gene.

### 2.7. Specimens and Immunohistochemistry

After the 2 weeks of UVB exposure, all mice were pentobarbital overdosed (200 mg/kg, IP) and 1 cm^2^ skin tissues were then quickly removed. The samples were fixed in 4% buffered neutral formalin solution for 24 hours at room temperature and paraffin embedded. Serial sections (7 *μ*m) were floated in warm water containing 2% gelatin to prevent peeling off of the sample. The sections were deparaffinized, rehydrated, and stained with hematoxylin and eosin (H&E). Examination was done by light microscopy at 200x magnifications.

HaCaT cells were fixed with 4% paraformaldehyde in 250 mM Hepes, pH 7.4, freshly diluted from 16% stocks stored at −20°C. After 5 minutes at room temperature, the cells were washed with phosphate-buffered saline and treated for 1 hour at 4°C with blocking solution (PBST, 1% FBS). Cells were incubated with Nrf-2 Ab in blocking solution overnight at 4°C in a wet chamber. After washing in PBST, the cells were incubated with appropriate secondary antibody mixtures in blocking solution for at least 1 hour at room temperature. The cells were washed three times in PBS and mounted in gold antifade reagent with DAPI (Molecular Probes, Eugene, OR). Confocal analysis was performed using a Leica TCS SP2 confocal microscope: 10 horizontal scans using a 63x (1.3 NA) oil immersion objective were recorded for each image with the imaging software (exported as a TIFF file).

### 2.8. Mouse Experiment

Animal experiments were conducted in accordance with International Standards on Animal Welfare and in accordance with the ethical standards of the Laboratory Animal Service Center of CMU (Affidavit of Approval of Animal Use Protocol Number 2016-259). Male ICR (BioLASCO Taiwan Co., Ltd.) mice were randomly divided into the following three groups (six mice in each group): control group treated with 0.1% DMSO culture medium, UVB-exposed group, and model group pretreated at dose of 200 *μ*g/mL and then exposed to UVB. The animals were housed (three animals per cage) in a temperature, humidity, and dark/light controlled colony. The animals were fed ad libitum with a standard laboratory diet and water. All mice, except the control group, were irradiated with the same UV source. Two 40-W UVB tubes (wavelength range: 290–320 nm; peak wavelength: 297 nm) constituted the UV source. The distance from the lamps to the animals' backs was 30 cm. The light intensity was measured with a UVB radiometer. The UVB irradiation was performed once a day and the mouse back was depilated before a day of the UVB radiation. The intensity of UVB was progressively increased by 100, 600, and 800 mJ/cm^2^ at day 1, day 5, and day 10, respectively. In the model group animal, extract of the* P. cicadae* was administered to UVB-irradiated mice backs at the day interval without UVB stimulation.

### 2.9. Statistical Analysis

A negative control (sham-irradiated cells) was included in all of the experiments. The results were expressed as the mean ± SD of at least three experiments. Statistical analysis of the control data versus treatments and UVB treatment was performed by Student's *t*-test (sigma plot 10.0.); ^*∗*^*p* < 0.05 was considered to be statistically significant; ^*∗∗*^*p* < 0.01; ^*∗∗∗*^*p* < 0.001.

## 3. Results

**Figure 1:**
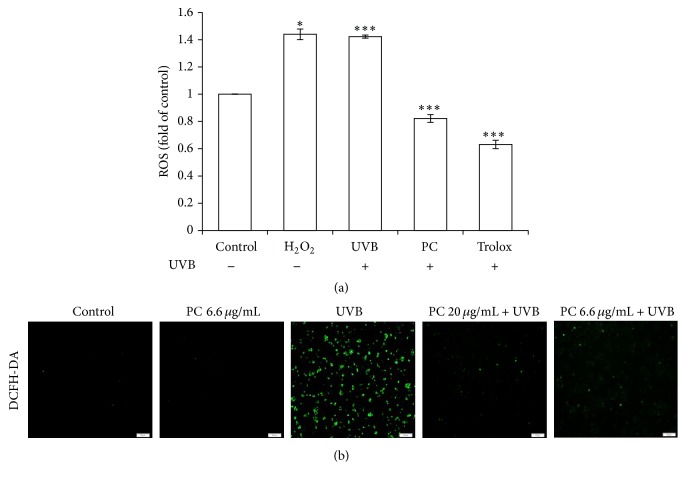
*P. cicadae* reduced the ROS production of HaCaT cells after UVB exposure. HaCaT cells were treated with 20 *μ*g/mL of* P. cicadae* for 4 hours, followed by exposure to 12 mJ/cm^2^ of UVB. The levels of ROS were measured by the DCFH-DA assay. (a) Fluorescence intensity of untreated control cells was set as 1 and results were expressed in X-fold. Trolox was used as an antioxidant control. H_2_O_2_ were used to generate hydroxyl radicals.* P. cicadae* effectively prevented UVB-produced ROS in HaCaT cells. (b) Representative immunofluorescent microscopy images, 40x magnification; scale bar = 500 *μ*m. ^*∗*^*p* < 0.05 was considered as a significant difference compared with the only UVB-irradiated group. ^*∗∗∗*^*p* < 0.001 was considered as a highly significant difference compared with the UVB-irradiated only group.

To investigate the effects of* P. cicadae* on the level of UVB-induced intracellular ROS, we cultured HaCaT cells with* P. cicadae* followed by UVB exposure, and the ROS level was measured by the DCFH-DA assay (Figure 1(a)). Hydrogen peroxide (H_2_O_2_), a cell membrane permeable compound and a precursor of various free radicals, was chosen as an oxidant whereas Trolox was used as antioxidant compound. The ROS level was dramatically increased in the UVB-irradiated and H_2_O_2_-treated cells. The UVB-induced ROS levels were significantly reduced in cells treated with* P. cicadae*. In comparison to the untreated control,* P. cicadae* alone had no effect on the generation of ROS. Plus, the higher doses (20 *μ*g/mL) of* P. cicadae* abolish more ROS formation than the lower dose (6.6 *μ*g/mL) of* P. cicadae* (Figure 1(b)). These results demonstrate that* P. cicadae* can effectively scavenge ROS induced by UVB on HaCaT cells.

**Figure 2:**
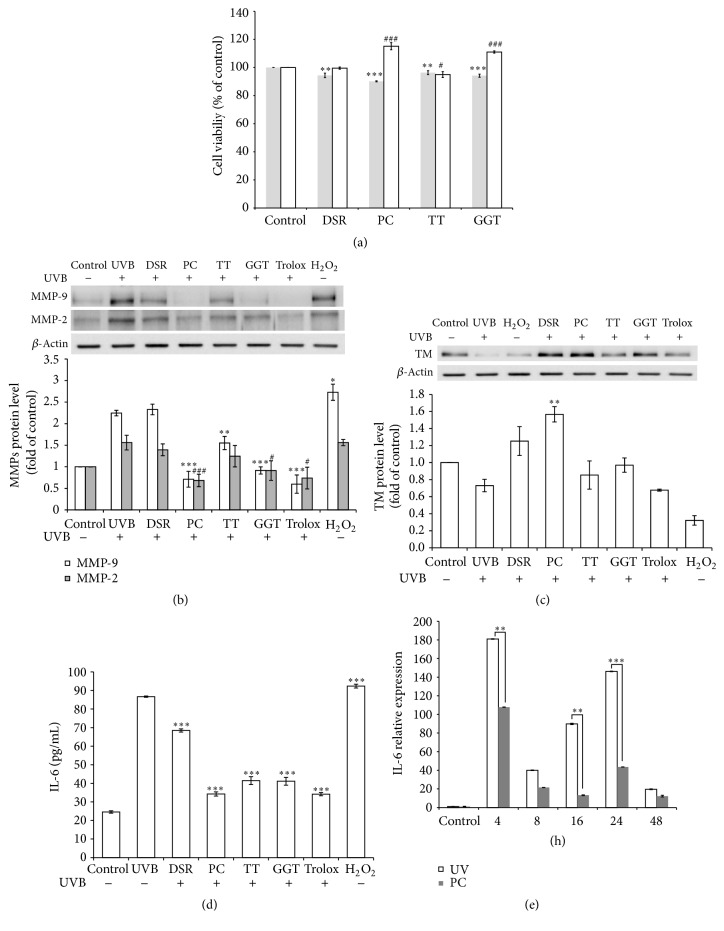
Effects of* P. cicadae* and herb extracts on MMP-9 and MMP-2 production in UVB-stimulated HaCaT cells. (a) Cell viability. HaCaT cells were exposed to extracts (gray bar) or added after UVB treated (white bar). For gray bar, *∗* stands for *p* < 0.05 versus control. For white bar, # stands for *p* < 0.05 versus control, ## stands for *p* < 0.01 versus control, and ### stands for *p* < 0.001 versus control. After 2 days of exposure, cell viability of the treated cells was examined by means of MTS assay (*n* = 5, mean ± SD). (b) The MMP-9 and MMP-2 levels were determined by Western blotting and *β*-actin was used as the loading control. The relative expression levels of MMP-9 (white bar) and MMP-2 (gray bar) were compared to control cells and presented as the mean ± SD for three independent experiments performed in triplicate. (c) The relative expression levels of TM were compared to control cells and presented as the mean ± SD for three independent experiments performed in triplicate. (d) IL-6 was quantified by ELISA in cell culture supernatants 24 hours following UVB and herb extracts (10 *μ*g/mL). Results are expressed as mean ± SEM of five independent experiments. (e) Relative IL-6 mRNA levels were measured by Q-PCR to 52 hours following UVB (20 *μ*g/mL). Histograms represent mean ± SEM of relative mRNA levels after normalization with 18S (*n* = 4-5 independent experiments). Trolox was used as an antioxidant control. H_2_O_2_ were used to generate hydroxyl radicals. DSR,* Divaricate saposhnikovia* root; TT, Tribulus ter; PC,* P. cicadae*; GGT, Ge Gen Tang. ^*∗*^*p* < 0.05 or ^#^*p* < 0.05, ^*∗∗*^*p* < 0.01 or ^##^*p* < 0.01, and  ^*∗∗∗*^*p* < 0.001 or ^###^*p* < 0.001 were considered as a significant difference compared to control group (^*∗*^*p* < 0.05 versus control).

To elucidate the defense mechanisms of* P. cicadae* on photooxidation, HaCaT cells were exposed to the UVB followed by incubation with* P. cicadae*.* Divaricate saposhnikovia* root, Tribulus ter, and Ge Gen Tang without cytotoxicity were chosen as compatible assays for their antioxidant capacity with* P. cicadae *(Figure 2(a)).* P. cicadae* showed the strongest activity against UVB-induced MMP-9 and MMP-2 expression at 24 hours (Figure 2(b)). The inhibitory effects of* P. cicadae* on MMPs production are similar to the effects of 10 *μ*M of Trolox, which is an established effective oxidative stress inhibitor. Our result further confirmed the TM downregulated after UVB exposure, whereas both* P. cicadae* and* D. saposhnikovia* root could effectively reverse the UVB-induced TM suppression [17]. Tribulus ter, Ge Gen Tang, and Trolox could also display mild activities and ameliorate the TM decreased by UVB treatment (Figure 2(c)). We next used IL-6 as a marker of activated keratinocytes. As shown in Figure 2(d), UVB challenge resulted in dramatic increases in IL-6 protein levels in HaCaT cells. In contrast, treatment with UVB plus* P. cicadae*,* D. saposhnikovia* root, Tribulus ter, or Ge Gen Tang significantly reduced IL-6 cytokine. Notably, the effectiveness of* P. cicadae* on IL-6 was comparable to Trolox. We further examined the effect of* P. cicadae* on IL-6 gene expression by real-time polymerase chain reaction. UVB exposure resulted in upregulated IL-6 gene at time point 4 hours, sustained to 48 hours. Treatment with* P. cicadae* markedly inhibited UVB-induced IL-6 mRNA expression (Figure 2(e)). These data indicated that* P. cicadae* might act to reduce cutaneous inflammation by inhibition of the mRNA and protein synthesis of IL-6.

**Figure 3:**
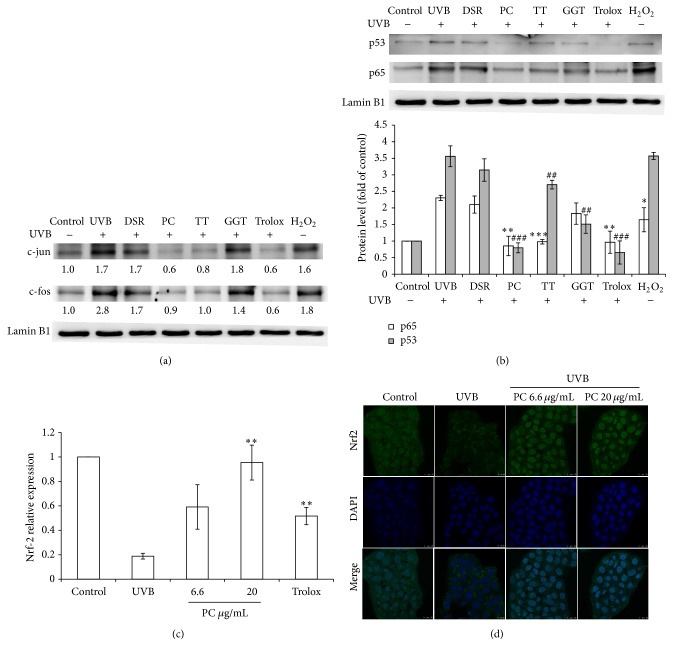
Effects of* P. cicadae* and herb extracts on UVB-stimulated transcription factors activated in HaCaT cells. (a) The p53 and p65 levels in cell nuclei were determined by Western blotting and lamin B1 was used as a loading control. The protein expression levels of p53 (gray bar) or p65 (white bar) were presented. For gray bar (p53), *∗* stands for *p* < 0.05 versus control. For white bar (p65), # stands for *p* < 0.05 versus control, ## stands for *p* < 0.01 versus control, and ### stands for *p* < 0.001 versus control. The relative expression levels of p53 (gray bar) and p65 (white bar) were compared to control cells and presented as the mean ± SD for three independent experiments performed in triplicate. (b) Relative Nrf-2 mRNA levels were measured by Q-PCRs following UVB and PC (20 *μ*g/mL). Histograms represent mean ± SEM of relative mRNA levels after normalization with 18S (*n* = 4-5 independent experiments). (c) Nrf-2 was revealed by green fluorescence and DAPI was revealed by blue fluorescence. Confocal microscope analysis was used to demonstrate the Nrf-2 accumulation in the nuclear fraction in HaCaT cells. (d) The c-jun and c-fos levels in cell nuclei were determined by Western blotting and lamin B1 was used as a loading control. The relative expression levels were compared to control cells and presented as the mean ± SD for three independent experiments performed in triplicate. Trolox was used as an antioxidant control. H_2_O_2_ were used to generate hydroxyl radicals. DSR,* Divaricate saposhnikovia* root; TT, Tribulus ter; PC,* P. cicadae*; GGT, Ge Gen Tang. ^*∗*^*p* < 0.05 or ^#^*p* < 0.05,  ^*∗∗*^*p* < 0.01 or ^##^*p* < 0.01, and ^*∗∗∗*^*p* < 0.001 or ^###^*p* < 0.001 were considered as a significant difference compared to control group (^*∗*^*p* < 0.05 versus control).

Since an activator protein- (AP-) 1 binding site was located in the upstream region of the IL-6 promoter, we further examined whether the c-jun and c-fos transcription factor was involved in the UVB-induced activation of the IL-6. The expression level of c-jun and c-fos was significantly enhanced by the UVB or oxidative stress. Consistent with this hypothesis,* P. cicadae* inactivated the c-jun and c-fos induced by UVB (Figure 3(a)). Since* P. cicadae* showed the ability to reduce UVB-induced inflammation and oxidative stress, we further investigated whether* P. cicadae* treatment decreased the transcription factors p53 and p65 activity in human keratinocytes. As shown in Figure 3(b), the expression levels of p53 and p65 in the nuclear fractionation were markedly induced with the UVB stimulation. However,* P. cicadae* reduced the UVB-induced activation of both p65 and p53—the inhibitory effect is comparable to Trolox. Tribulus ter could only suppress UVB-induced p53 but not on p65. These findings showed* P. cicadae* participates in the antioxidation and anti-inflammation induced by UVB and reversed the nuclear translocation of p53 and p65.

We examined the effects of* P. cicadae* on the activation of master regulator of antioxidant response Nrf2. Results in Figure 3(c) reveal that relative mRNA expression of transcription factor Nrf2 is significantly induced following treatment with* P. cicadae*, while the UVB significantly reduced the mRNA relative level of Nrf2. HaCaT cells exposed to UVB showed retention of Nrf-2 in cytosol, whereas this fluorescence was substantially translocated to nucleus by* P. cicadae* (Figure 3(d)). The protective effects of* P. cicadae* against UVB were directly evidenced by contribution of the antioxidant defense system.

**Figure 4:**
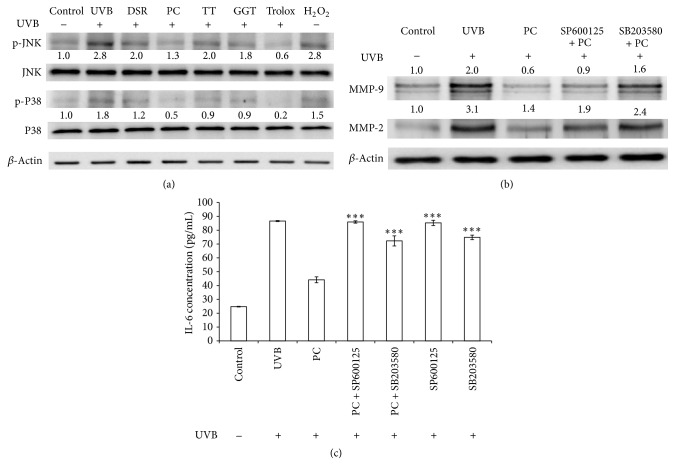
*P. cicadae* inhibits UVB-induced MMPs and IL-6 via suppression of JNK MAPK in HaCaT cells. (a) The JNK and p38 levels in cell nucleus were determined by Western blotting and *β*-actin was used as loading controls. The relative expression levels of JNK and p38 were compared to control cells and presented as the mean ± SD for three independent experiments performed in triplicate. (b) The expression of MMPS in the presence of SP600125 and SB203580, respectively. MMPs levels in cell were determined by Western blotting and *β*-actin was used as a loading control. The relative expression levels of MMP-9 and MMP-2 were compared to control cells and presented as the mean ± SD for three independent experiments performed in triplicate. (c) Effects of* P. cicadae* on the IL-6 in the presence of SP600125 and SB203580, respectively. The UVB-incubated HaCaT cells pretreated with signal inhibitors were incubated with* P. cicadae* for 24 hours. The production of IL-6 was analyzed by ELISA. The data represent at least three independent experiments. ^*∗*^*p* < 0.05,  ^*∗∗*^*p* < 0.01, and  ^*∗∗∗*^*p* < 0.001 were considered as a significant difference compared to control group (^*∗*^*p* < 0.05 versus control).

The UVB irradiation resulted in phosphorylation of the stress-activated MAP kinases p38 and JNK has been reported. Cells added with* P. cicadae* exhibited decreased phosphorylation of JNK and p38 compared to cells treated only with UVB (Figure 4(a)). The same pattern was evident using two kinase inhibitors. The results showed that* P. cicadae* reduced the inflammation activity of UVB-stimulated HaCaT cells through p38 and JNK signaling, which could subsequently decrease IL-6 production. Cells pretreated with SB203580 (p38 inhibitor) before stimulation with* P. cicadae* displayed decreased expression of MMPs compared to cells treated only with* P. cicadae. *Furthermore, blocking p38 and JNK increased the IL-6 content attenuated by* P. cicadae* (Figure 4(b)). The data demonstrated that the* P. cicadae*-induced anti-inflammatory effect may be mediated by activation of the p38 and JNK pathway.

**Figure 5:**
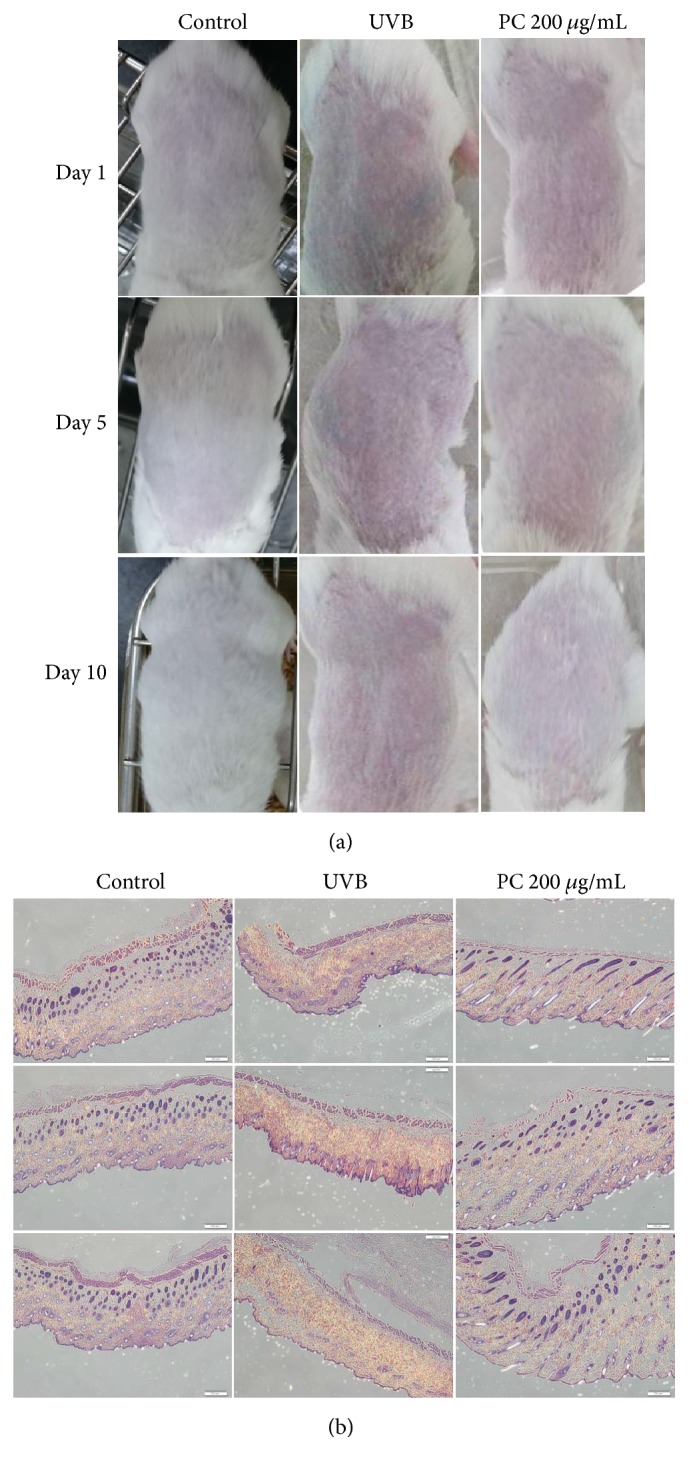
Effect of topical treatment of mouse skin with* P. cicadae* on UVB-induced damage. (a) ICR mice skin was treated with either 200 *μ*g/mL* P. cicadae* or PBS and was exposed to UVB every 5 days, as described in Methods. Mouse skin treated topically with* P. cicadae* before UVB exposure reduced UVB-induced signs of sunburn tissue damage. (b) Skin biopsies were processed by H&E staining by a routine procedure. Micrographs show representative findings of skin in response to UVB, sampled after UVB exposure, and a representative section of H&E staining from three independent experiments is shown. Pretreatment of* P. cicadae* inhibited UVB-induced extensive structural damage in both epidermis and dermis (magnification, 40x).

Mouse dorsal skin physiological changes were recorded after treatment with* P. cicadae* and UVB irradiation for 10 consecutive days. In our study, prolonged skin exposure to UVB leads to skin inflammation erythema, the skin sebum content was significantly reduced, and the loss of skin fullness led to wrinkling and aged appearance of the skin. Pretreatment with* P. cicadae* 1 hour before each UVB exposure inhibits these inflammatory reactions in the mice skin. The* P. cicadae* group decreased UVB-induced erythema (Figure 5(a)). In addition, the* P. cicadae* treatment group (at the dose of 200 *μ*g/mL) showed the most remarkable effect among all groups. To analyze the damage induced by UVB and senescence, Figure 5(b) shows H&E-stained images of mouse skin. Histopathological observations indicated an intact structure of the whole skin with no treatment. Prominent epidermal hyperplasia with abnormal keratinization and hyperkeratosis were observed in the upper epidermis of UVB group. Beneath the epidermis, a clear disappearance of dermal papillae was also found in UVB treatment. Pretreatment with* P. cicadae* almost completely prevented UVB-induced damage in epidermis. In addition, dermal-epidermal junction returned to near-normal levels as compared to that of the control group because of the well-marked appearance of dermal papillae and epidermal rete ridge. Moreover, the upper portion of the dermis showed an order arrangement of hair follicles. These results indicated that* P. cicadae* alleviate the UV-induced photodamage.

## 4. Discussion

A wide variety of phenylalkylketone compounds derived from natural products activate potent antioxidant activities in skin [31]. In this study, we investigated the UVB-protection activity of clinically used herbal medicines on skin disorder AD.* P. cicadae* showed significant skin protection effects, including inhibition of expression of IL-6 and MMPs; downregulation of the irradiation-induced p53, AP-1, and NF-*κ*B; and upregulation of the amount of Nrf-2. These results suggest that the* P. cicadae* decreased inflammation induced by UVB, which could be achieved via depletion of ROS and its downstream signaling mechanisms. Two cicadamide N-acetyldopamine compounds identified as* P. cicadae* major components have been reported to have anti-inflammatory efficacy [32]. A previous report has shown that fractionation from the methanolic extracts of* P. cicadae* exerts the antioxidant function by direct interaction with generated radicals or inhibition of radical generation [4], which is consistent with lower levels of ROS in our results and may contribute to the dermatoprotective activity.

In this study,* P. cicadae* displays the strongest anti-UVB effects among the popular TCM treatments for AD.* P. cicadae* clearly stimulated the nuclear translocation of Nrf-2 and inhibited the synthesis of UVB-induced inflammatory mediators including IL-6, MMP-2, and MMP-9. In addition,* P. cicadae* treatment further enhanced UVB-mediated protein expression of p53. The antioxidative activity from* P. cicadae* crude extract was comparable to 20 *μ*g/mL of antioxidant Trolox on UVB-irradiated HaCaT cells [33]. It is established that AD skin shows dysregulation of Th2 cytokines as well as alterations in lipid composition of the stratum corneum that are critical in maintaining the permeability barrier of the epidermis [34, 35]. These results could be of value for development of* P. cicadae* extract as a skin care agent that is worthy of further clinical investigations. We should, however, particularly take into account the rarity of species, and the possible allergic reactions linked to the administration of the animal-based remedies [36–38]. However,* P. cicadae* is the cast-off shell byproduct of the cicada and the use could be done in a sustainable way. The application of the insect medicinal products would not endanger the survival of this insect species.

Previous studies have shown that UVB activated the MAPK pathways in human skin cells [39]. We also found that inhibitors of p38 or JNK do in fact block the effect of* P. cicadae* to inhibit IL-6 synthesis and MMP-2 in HaCaT cells. Thus,* P. cicadae* is an effective suppressor of inflammation caused by UVB via p38 and JNK activation and by the subsequent downregulation of NF-*κ*B and AP-1 production. The promoters of various genes coding for proteins involved in inflammatory processes harbor specific DNA sequences onto which the proinflammatory transcription factors NF-*κ*B and/or AP-1 can bind. We analyzed the 5′ flanking region of the human promoter sequence by performing TFSEARCH software (http://www.cbrc.jp/research/db/TFSEARCH.html). The promoter regions of IL-6 and MMP-2 have the transcriptional factors NF-*κ*B and AP-1 binding sites [40–42]. AP-1 and NF-*κ*B upregulate the transcriptions of several MMPs. It is known that UV irradiation causes extracellular matrix degradation via the induction of transcription factor AP-1 and subsequent increases in the production of MMPs in human skin. Plus, UVB stimuli drive gene transcription of the cytokine IL-6, which has been implicated not only in immune regulation but also in pleiotropic action including endocrine and aging. Therefore, this study provides the molecular mechanisms of the antioxidative and anti-inflammatory activities of* P. cicadae* in human skin cells.

**Figure 6:**
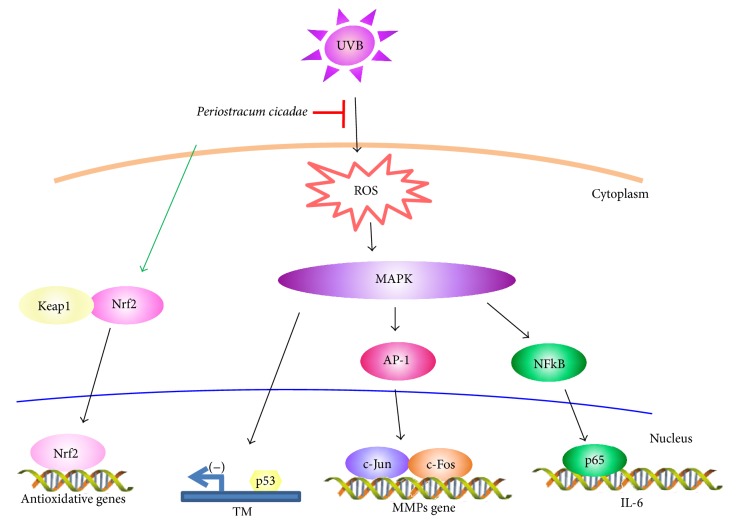
Proposed mechanism by which* P. cicadae* abolished UVB-stimulated inflammation. Cartoon shows that* P. cicadae* decreases expression of MMPs, TM, and IL-6 in HaCaT cells. The suppressive effect of* P. cicadae* on photodamage was induced through JNK and p38 activation.* P. cicadae* suppression of the JNK or p38 pathway could diminish the UVB-induced AP-1, p53, and p65 activation. Moreover,* P. cicadae*-induced Nrf-2 activation and translocation from the cytosol to the nucleus.

The inhibition of MMP expression has been reported to improve UV-induced photoaging in terms of protection from collagen degradation [43]. To verify the mouse model as a photodamage model, we confirmed that it exhibited chronic photodamage symptoms such as erythema and desquamation in the dorsal skin. We successfully prevented UV radiation-induced cutaneous photodamage in mice by topical administration of* P. cicadae*. These data showed that* P. cicadae* downregulates MMPs protein expression which provides strong support for the clinical efficacy. In summary, this study demonstrates that* P. cicadae* administration protects against UVB-induced chronic photodamage through antioxidant, anti-inflammatory, and signaling regulatory effects in HaCaT cells and the skin of hairless mouse (Figure 6). Hence, we propose that* P. cicadae* may be useful as an antiphotoaging agent.

## 5. Conclusion

This is the first report on the action mechanisms of the* P. cicadae* extract that protect skin cells against oxidative insult by modulating ROS concentration, IL-6, MMPs generation, antioxidant enzymes activity, and cell signaling pathways. The present study concluded that* P. cicadae* attenuates the expression of IL-6, MMP-2, and MMP-9 in UVB-treated HaCaT cells. The extract abrogated UVB-induced activation of NF-*κ*B, p53, and AP-1. The downmodulation of IL-6 by* P. cicadae* extract was inhibited by the p38 inhibitor or JNK inhibitor. Further, the extract attenuated the expression of NF-*κ*B as well as induced thrombomodulin in keratinocytes and then effectively downregulated inflammatory responses in the skin. The nuclear accumulation and expression of Nrf2 were increased by* P. cicadae *treatment. Treatment with* P. cicadae* remarkably ameliorated the skin's structural damage induced by irradiation. Hence, the* P. cicadae* extract could be used as a novel dermatological anti-inflammation agent in skin care products.
